# Manual centile-based early warning scores derived from statistical distributions of observational vital-sign data^☆^

**DOI:** 10.1016/j.resuscitation.2018.06.003

**Published:** 2018-08

**Authors:** Peter J. Watkinson, Marco A.F. Pimentel, David A. Clifton, Lionel Tarassenko

**Affiliations:** a Nuffield Department of Clinical Neurosciences, Oxford University Hospitals NHS Trust, OX3 9DU Oxford, UK; b Institute of Biomedical Engineering, Department of Engineering Science, University of Oxford, OX3 7DQ Oxford, UK

**Keywords:** Physiological monitoring, Vital signs, Early warning system, Risk scoring systems, Medical emergency team

## Abstract

**Aims of study:**

To develop and validate a centile-based early warning score using manually-recorded data (mCEWS). To compare mCEWS performance with a centile-based early warning score derived from continuously-acquired data (from bedside monitors, cCEWS), and with other published early warning scores.

**Materials and methods:**

We used an unsupervised approach to investigate the statistical properties of vital signs in an in-hospital patient population and construct an early-warning score from a “development” dataset. We evaluated scoring systems on a separate “validation” dataset. We assessed the ability of scores to discriminate patients at risk of cardiac arrest, unanticipated intensive care unit admission, or death, each within 24 h of a given vital-sign observation, using metrics including the area under the receiver-operating characteristic curve (AUC).

**Results:**

The development dataset contained 301,644 vital sign observations from 12,153 admissions (median age (IQR): 63 (49–73); 49.2% females) March 2014–September 2015. The validation dataset contained 1,459,422 vital-sign observations from 53,395 admissions (median age (IQR): 68 (48–81), 51.4% females) October 2015–May 2017. The AUC (95% CI) for the mCEWS was 0.868 (0.864–0.872), comparable with the National EWS, 0.867 (0.863–0.871), and other recently proposed scores. The AUC for cCEWS was 0.808 (95% CI, 0.804–0.812). The improvement in performance in comparison to the continuous CEWS was mainly explained by respiratory rate threshold differences.

**Conclusions:**

Performance of an EWS is highly dependent on the database from which itis derived. Our unsupervised statistical approach provides a straightforward, reproducible method to enable the rapid development of candidate EWS systems.

## Introduction

Early warning score (EWS) systems based on vital signs rely on data-driven approaches to derive the threshold values for the scores assigned to each physiological variable [[1], [2], [3], [4]]. While most of these systems are based on a large database of vital signs *collected manually*, we previously used a large dataset of *continuously-recorded* vital-sign data to derive our original centile-based EWS (CEWS) [2]. For the latter, a large dataset comprising 64,622 h of vital-sign data acquired from over 800 acutely ill in-hospital patients connected to bedside monitors was used to investigate the statistical distributions of each vital sign. From this, an aggregate centile-based alerting system with seven bands of risk level for each vital sign was designed (i.e., scores of 0, 1, 2 and 3, as used in other systems [1,3,4]). Observations are then treated as being “abnormal” if they occur at the extremes of the distributions of the vital signs; i.e., the thresholds for low values were set to the values that correspond to the 10th, 5th, and 1st centiles and the thresholds for high values were set to be the integer values that correspond to the 90th, 95th, and 99th centiles, for scores of 1, 2, and 3, respectively. The threshold values in the centile-based EWS derived using this approach, with continuously-monitored data, are different from those in other EWS systems. In particular, the thresholds for high values of respiratory rate (RR) are markedly different [2].

Several studies have reported fundamental differences between continuously-acquired and manually-collected vital-sign data in hospital settings [[5], [6], [7], [8], [9], [10]]; it is therefore not surprising that our original centile-based EWS differed from other systems designed using vital-sign values collected manually. In this paper, we investigate the changes in threshold values for each vital sign in a centile-based system when the centiles are derived from manually-recorded data. We then report the performance of the manual CEWS system in comparison to other early warning scores on a large database of vital-sign data collected from in-hospital patients.

## Methods

Approval from the Health Research Authority was obtained for this study from the Oxford Research Ethics Committee (REC reference: 16/SC/0264).

### Data collection

A database of vital sign observations was collected from adults (aged at least 16 years) admitted to the Oxford University Hospitals NHS Trust on or after 10 March 2014 and discharged on or before 01 May 2017. Clinical staff recorded patients’ vital signs at the bedside on general wards using our System for Electronic Notification and Documentation (SEND) [11,12]. The following data were recorded: date and time of observation (automatically by SEND); the manual measurement of heart rate, systolic and diastolic blood pressure, respiratory rate, body temperature, neurological status using the Alert-Verbal-Painful-Unresponsive (AVPU) scale, peripheral oxygen saturation (SpO_2_); and a record of whether or not supplemental oxygen support was given to the patient at the time of the measurement. This does not include observations carried out while patients were in the ICU. The database also contains administrative and patient demographic information, and occurrences of patient death, cardiac arrests, unanticipated ICU admissions, and their corresponding timings, which were identified from the patient administration system and the ICU clinical information system.

All admissions to four Oxford organisation hospitals: the John Radcliffe Hospital – a large university hospital, the Horton General Hospital – a small district general hospital, the Churchill Hospital – a large university cancer centre, and the Nuffield Orthopaedic Hospital, were considered for this study. Only adult (aged at least 16 years) admissions where, at least, one complete set of vital signs recorded electronically, were considered for inclusion in the analysis. Where the specialty on admission to the hospital was palliative medicine, the admission was excluded from the analysis. Data from patients who were discharged alive from hospital before midnight on the day of admission were excluded.

Two datasets were extracted from this database. The first dataset, the “development” dataset, includes 301,644 vital sign observations collected from 12,153 admissions (median age (IQR): 63 (49–73); 49.2% females) between 24 March, 2014 and 30 September, 2015 (see Table 1). This dataset was used to estimate new threshold values for each individual vital sign using our centile-based approach, but this time using manually-recorded measurements (instead of automatically-measured values). We used a second (“validation”) dataset that includes clinical data from emergency admissions between 1 October, 2015 and 1 May, 2017 to evaluate the performance of our EWS system. This second dataset comprises 1,459,422 vital-sign observations from 53,395 admissions, median age (IQR) 68 (48–81), 51.4% females (as detailed in Table 1). Data pre-processing was carried out before evaluation. Vital-sign sets for which more than two measurements were absent or which were physiologically-implausible (i.e., recorded in error) were excluded. For those observation sets with one or two missing measurements, the missing value was replaced by the population mean value of the corresponding measurement (from the development dataset). We note that by replacing the missing value with the population mean value, we make the assumption that this measurement does not contribute to the overall early warning score, because the score assigned to this particular variable will be 0.

**Table 1:** Demographic descriptors for admissions included in both development and validation datasets. The Charlson Comorbidity Index and definitions of surgical specialties were determined according to the methodology and specification provided by NHS Digital. Continuous variables are shown with Median (IQR) [Mean]. IQR refers to the interquartile range.

	Development set	Validation set
Admissions:		
No. of admissions	12,153	53,385
Age (years)	63 (49–73) [61]	68 (48–81) [64]
Females, No. (%)	5976 (49.2)	27,433 (51.4)

Ethnic category, No. (%)		
Asian or Asian British	274 (2.3)	1283 (2.4)
Black or Black British	158 (1.3)	538 (1.0)
Mixed	118 (1.0)	362 (0.7)
White	9,035 (74.3)	43,017 (80.6)
Other Ethnic Groups	2,539 (20.9)	7636 (14.3)
Not disclosed	29 (0.2)	559 (1.0)
Length of stay (days)	2.4 (0.8–7.1) [7.2]	3.1 (1.3–8.0) [7.0]
Surgical admissions, No. (%)	5259 (43.3)	20,412 (38.2)
Charlson Comorbidity Index	3 (0–10) [5.4]	3 (0–12) [6.4]

Outcomes studied, No. (%)		
Composite outcome	548 (4.5)	3507 (6.6)
In-hospital mortality	358 (3.0)	2805 (5.2)
Unanticipated ICU admission	225 (1.8)	907 (1.7)
Cardiac arrest	22 (0.2)	173 (0.3)

Observation sets:		
No. of observations	301,644	1,459,422
Heart rate, beats per minute	82 (71–93) [83]	80 (70–92) [82]
Respiratory rate, breaths per minute	17 (16–18) [17]	18 (16–18) [17]
SpO_2_, %	97 (95–98) [96]	96 (95–98) [96]
Systolic blood pressure, mmHg	123 (110–138) [125]	125 (111–142) [128]
Temperature, °C	36.3 (36.0–36.7) [36.4)	36.4 (36.0–36.7) [36.4]

AVPU level, No. (%)		
Alert	296,221 (98.2)	1,417,719 (97.1)
Responds to Voice	4,524 (1.5)	31,662 (2,2)
Responds to Pain	603 (0.2)	6613 (0.5)
Unresponsive	296 (0.1)	3428 (0.2)
Supplemental Oxygen Support, No. (%)	53,693 (17.8)	265,759 (18.2)

Charlson Comorbidity Index guidelines are available at https://beta.digital.nhs.uk/publications/ci-hub/summary-hospital-level-mortality-indicator-shmi, and the definitions of surgical specialties are available at https://www.datadictionary.nhs.uk/data_dictionary/attributes/m/main_specialty_code_de.asp (both accessed in September 2017).

### Methods: estimation of threshold values from vital-sign database

**Fig. 1:**
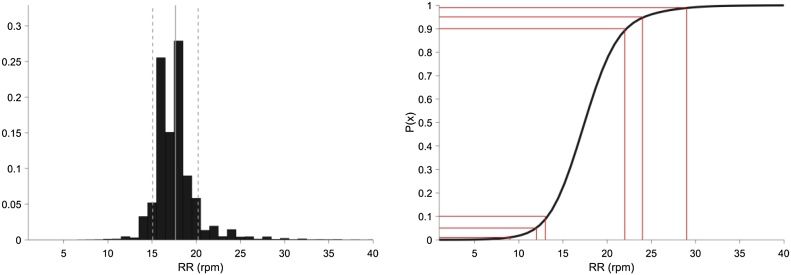
Representation of the normalised histogram (left), and cumulative distribution functions (cdf), P(x) for respiratory rate, computed from the development dataset, which includes manual measurements of respiratory rate collected electronically from patients admitted to the hospital. For the plot on the left, the central vertical line indicates the mean of the data, with the two vertical (dashed) lines either side corresponding to one standard deviation. For the plot on the right, the 1st, 5th, 10th, 90th, 95th and 99th centiles are shown on the vertical axis and the corresponding threshold values on the horizontal axis.

Using the development dataset of observation sets (which includes manual measurements of vital signs), and using the same unsupervised, statistical approach proposed by Tarassenko et al. [2], we obtained new values for the lower and upper thresholds for each vital sign (except for SpO_2_). To maximise the use of the dataset for robust estimation of the centiles in the tails of the distributions, a smooth estimate of the distribution of each vital sign was obtained using a kernel-based density estimator. The bandwidth, h, of the Gaussian kernels was computed using the normal distribution approximation, given by $h=1.06\hat{\sigma }n^{-1/5}$, where $\hat{\sigma }$ is the standard deviation of the n samples [13]. Using the resulting (smoothed) cumulative distribution of each vital sign estimated from the development dataset, the lower threshold values were then set to the integer values that correspond to the 10th, 5th, and 1st centiles and the upper threshold values were set to be the integer values that correspond to the 90th, 95th, and 99th centiles (as shown in Fig. 1), for scores of 1, 2, and 3, respectively. In other words, a score of 3 is generated when a vital sign is below the 1st centile or above the 99th centile, a score of 2 corresponds to the vital sign being between the 1st and 5th centiles or between the 95th and 99th centiles, and a score of 1 corresponds to the vital sign being between the 5th and 10th centiles or between the 90th and 95th centiles. For SpO_2_, with a one-sided distribution, the lower threshold values were set to the integer values that correspond to the 20th, 10th and 2nd centiles for scores of 1, 2, and 3, respectively (see Supplemental Materials–Appendix A). For temperature, given the very small ranges of possible values between the lower and upper centiles, the lower threshold values were set to the values with one decimal place that correspond to the 10th and 1st centiles and the upper threshold values were set to the values with one decimal place that correspond to the 90th and 99th centiles, for scores of 1 and 3 respectively. A further modification to this scoring system was evaluated: the use of an additional score of 2 if the patient was given supplemental oxygen support at the time of the measurement, as with the NEWS scoring system [3].

### Performance assessment

We assessed EWS performance using the validation dataset of vital signs (independent of the development set used to calculate the manual CEWS threshold values). We evaluated the discriminative ability using the composite outcome of cardiac arrest, unanticipated intensive care unit (ICU) admission, or death (categorised as major adverse events), each within 24 h of a given vital-sign observation, as performed in previous studies [1,3,4]. Where there were multiple outcomes (for example, cardiac arrest followed by unanticipated ICU admission), only the first event to occur was used for analysis. Hence, observation sets performed after the first event were excluded from this analysis. The number of patient admissions with a combined outcome in the validation set was 3,688, approximately 7% of the total number of admissions (see Table 1). We have also evaluated the performance of the scoring systems for each of the three individual outcomes.

We first assessed the performance of the continuous CEWS system [2] on the validation dataset. To determine which vital signs contribute to differences in performance of the manual CEWS, we then assessed the effect of substituting the manually-derived threshold values of each vital sign in turn (keeping the continuously-derived threshold values of the remaining vital signs the same). Performance was assessed using the area under the receiver-operating characteristic curve (AUC) [14] for the composite outcome, by determining the percentage change, as such:

$$\frac{{AUC}_{CEWS,i}-AUC_{CEWs}}{AUC_{CEWS}}\times 100\;(\%)$$

where $AUC_{CEWS}$ is the performance of the continuous centile-based EWS, and $AUCROC_{CEWS,i}$ corresponds to the AUC of the continuous centile-based EWS system with the new manual thresholds substituted for each of the vital signs, *i*, in turn.

We then evaluated the performance of the manual centile-based EWS system (with the new threshold values for all vital signs and the additional score of 2 for use of oxygen support) and compared it with that of other EWS systems published in the literature. In addition to the AUC metric, we determined the area under the precision-recall curve (AUC-PR), as suggested in an increasing body of literature [[15], [16], [17]]. The precision-recall curve is a plot of the sensitivity (recall) against the positive predictive value (PPV, or precision), a metric which is associated with the operational consequences and workload/clinical burden imposed on clinicians by alerting systems [16].

We also evaluated the performance (as given by the AUC) of the EWS systems using subsidiary-derived outcome variables that include the occurrence of the composite adverse event within 12 or 48 h of an observation set.

## Results

The characteristics of the vital signs in our development and validation datasets are shown in Table 1. Fig. 1 shows an example of the histogram (normalised such that the area-under-the-curve is 1.0) and of the cumulative distribution function for manually-collected respiratory rate (see Supplemental Materials–Appendix A for the equivalent figures for the other vital signs, including those for continuously-acquired vital signs, which are based on our previous results [2]). The differences between the scores for the continuous CEWS and its manual version are shown in Table 2.

**Table 2:** Range of values for each weighting for the vital signs in the centile-based EWS derived from continuously-acquired vital-sign data using bedside monitors, and for the manual centile-based EWS system. *Inspired O_2_, denoting use of any oxygen support, was investigated as a separate addition to the EWS system.

Continuous Centile-based EWS [2]							
	Score						
Variable	3	2	1	0	1	2	3
Heart rate	≤ 50	51–58	59–63	64–104	105–112	113–127	≥ 128
Respiratory rate	≤ 7	8–10	11–13	14–25	26–28	29–33	≥ 34
Temperature	≤ 35.4		35.5–35.9	36.0–37.3	37.4–38.3		≥ 38.4
Systolic BP	≤ 85	86–96	97–101	102–154	155–164	165–184	≥ 185
SpO_2_	≤ 84	85–90	91–93	≥ 94			
AVPU scale				A	V		P, U

Manual Centile-based EWS							
	Score						
Variable	3	2	1	0	1	2	3
Heart rate	≤ 42	43–49	50–53	54–104	105–112	113–127	≥ 128
Respiratory rate	≤ 7	8 – 11	11 – 12	13 – 21	22–23	24–28	≥ 29
Temperature	≤ 35.4		35.5–35.9	36.0–37.3	37.4–38.3		≥ 38.4
Systolic BP	≤ 83	84–90	91–100	101–157	158–167	168–184	≥ 185
SpO_2_	≤ 84	85–90	91–93	≥ 94			
Inspired O_2_*				Air		Any O_2_	
AVPU scale				A	V		P, U

**Fig. 2:**
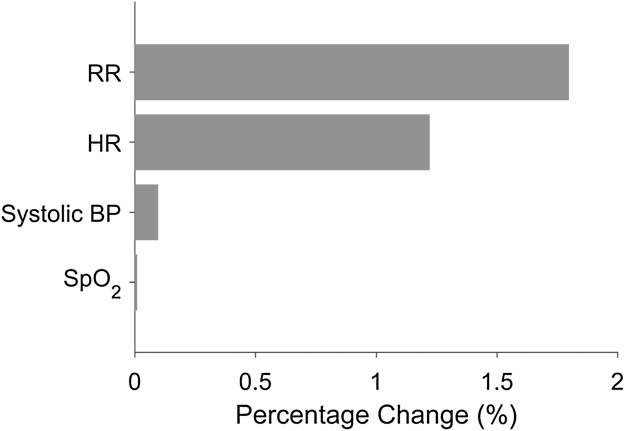
Percentage change (%) in performance (as given by the AUC) of the continuous CEWS when the thresholds for each vital sign are replaced by the equivalent thresholds derived from the distribution of manually-recorded measurements, with the threshold values for the other four vital signs remaining unchanged.

Fig. 2 shows the change in performance (as measured by the AUC) for the continuous centile-based EWS when the thresholds for each vital sign in turn are replaced by the equivalent thresholds derived from the manual dataset. Changing the thresholds for RR produces a percentage change of approximately 2% in the performance of the original, continuous centile-based EWS system.

The performance of the different EWS systems on the validation dataset is shown in Table 3. Our continuous CEWS has an AUC of 0.808 (95% CI, 0.804–0.812) for the validation dataset using the composite outcome of major adverse event within 24 h of an observation set. The manual CEWS without supplemental oxygen has an AUC of 0.836 (0.832–0.840). When supplemental oxygen is included, the manual CEWS has an AUC of 0.868 (0.864–0.872), which is comparable to that of NEWS, 0.867 (0.863–0.871). The same trend (increasing performance) is observed if we consider the AUC-PR values. The AUC (95% CI) for the other EWSs using the composite outcome ranged from 0.768 (0.764–0.773) [31] to 0.865 (0.861–0.869) [4] (the ROC and Precision-Recall curves for all EWSs are represented in Supplemental Material – Appendix B). The results of the evaluation of the EWSs using each individual outcome are described in Supplemental Material–Appendix C.

**Table 3:** Area under the receiver-operating characteristics curve (AUC) and area under the precision-recall curve (AUC-PR), and corresponding 95% confidence interval (CI) for the Manual centile-based EWS (CEWS) and 22 other early warning score (EWS) systems, using cardiac arrest, unanticipated admission to ICU or death within 24 h of the observation set as the (composite) outcome. The EWS number (EWS no.) refers to those used in other figures and sections of the manuscript. Results are presented in descending order of AUC, with the results relating to the scores that use the methodology discussed in the manuscript highlighted. [*] indicates systems that have an additional score for supplemental oxygen support.

EWS no.	EWS	AUC	95% CI	AUC-PR	95% CI
–	Manual CEWS [*]	**0.868**	**0.864–0.872**	**0.161**	**0.155–0.167**
21	NEWS [3] [*]	0.867	0.863–0.871	0.163	0.157–0.169
22	Badriyah et al. [4] [*]	0.865	0.861–0.869	0.157	0.151–0.162
19	Lilienfeld-Toal et al. [34] [*]	0.860	0.857–0.864	0.151	0.146–0.157
8	Goldhill et al. [25]	0.846	0.842–0.850	0.148	0.142–0.153
18	Lilienfeld-Toal et al. [34]	0.846	0.842–0.850	0.143	0.137–0.148
11	Paterson et al. [28]	0.843	0.839–0.847	0.159	0.153–0.164
–	**Manual CEWS**	**0.836**	**0.832–0.840**	**0.140**	**0.135–0.146**
9	Chatterjee et al. [26]	0.827	0.823–0.831	0.124	0.118–0.129
7	Allen [24]	0.826	0.822–0.830	0.130	0.125–0.135
1	Wright et al. [18]	0.823	0.818–0.827	0.129	0.124–0.135
4	Cooper et al. [21]	0.823	0.818–0.827	0.129	0.124–0.134
5	Subbe et al. [22]	0.822	0.818–0.826	0.130	0.125–0.136
2	Subbe et al. [19]	0.821	0.817–0.825	0.126	0.120–0.131
3	Riley et al. [20]	0.821	0.817–0.825	0.123	0.118–0.128
12	Smith et al. [35]	0.821	0.817–0.825	0.130	0.124–0.135
10	Andrews et al. [27]	0.818	0.813–0.822	0.131	0.126–0.137
14	Gardner-Thorpe et al. [30]	0.817	0.813–0.822	0.130	0.125–0.136
16	Odell [32]	0.817	0.813–0.822	0.127	0.122–0.133
13	Lam et al. [29]	0.817	0.813–0.822	0.130	0.125–0.136
6	Rees et al. [23]	0.817	0.813–0.821	0.128	0.122–0.133
17	Hancock et al. [33]	0.815	0.811–0.820	0.128	0.123–0.133
**20**	**Continuous CEWS** [2]	**0.808**	**0.804–0.812**	**0.128**	**0.123–0.133**
15	Subbe et al. [31]	0.768	0.764–0.773	0.101	0.096–0.106
–	CART, Churpek et al. [39]	0.729	0.725–0.734	0.098	0.092–0.104

**Fig. 3:**
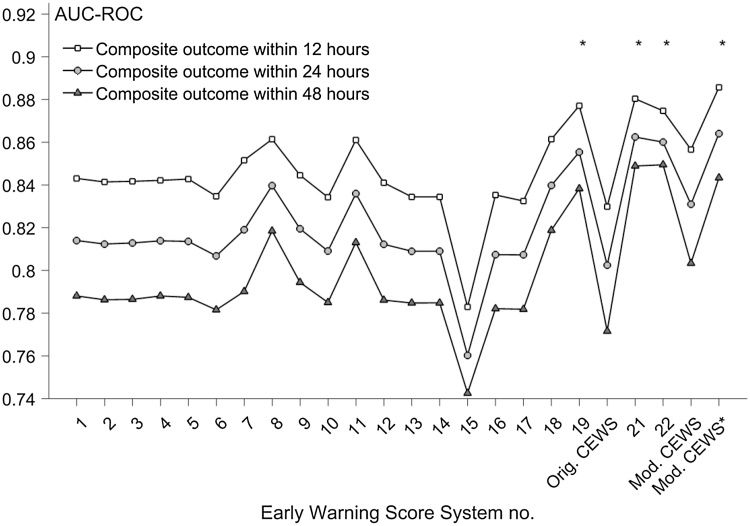
AUC values for the manual CEWS and another 22 EWS systems, for the composite outcome of cardiac arrest, unanticipated ICU admission, or death within {12, 24, and 48} h of a given observation set. The system numbers (horizontal axis) correspond to those used in Table 3. The symbol ‘*’ denotes the systems that have an additional score for supplemental oxygen support.

Fig. 3 shows the performance of the different scoring systems using the composite outcome of a major adverse event occurring within *T* = 12, 24, or 48 h of an observation set. The change in performance with respect to *T* is consistent for all scoring systems, deteriorating as T increases.

## Discussion

Our original centile-based EWS system was developed using vital-sign data acquired continuously from bedside monitors. We used a statistical approach to determine the threshold values for each physiological variable from which scores are calculated. In this study, we applied the same statistical approach using vital-sign data acquired manually by clinical staff, the current method of recording vital signs in clinical practice on general wards. The improvement in performance between continuous CEWS and our new manual CEWS was mainly explained by differences in respiratory rate thresholds. Although the median value for respiratory rate acquired manually from hospitalised patients is similar to that in continuous data acquired from patient monitors, the distributions, especially in the tails, are very different (see Supplemental Materials: Appendix A), hence the threshold values are also different, especially for the upper thresholds for respiratory rate (tachypnoea). With these thresholds, the manual CEWS has superior discriminative performance as measured by the AUC metric. On a separate, validation dataset, manual CEWS, an unsupervised centile-based approach to EWS development produces values of AUC at least as high as supervised methods, such as NEWS, in which knowledge of patient outcome is used during development.

The difference in performance between continuous and manual CEWS suggests that the database with which an EWS is developed affects performance. The key difference between the two training databases is that, with continuous CEWS, the vital signs were (continuously) electronically recorded. Previous studies [6,8] have shown discrepancies between clinical measurements by clinical staff and electronic monitors.

We also found a clear effect associated with the inclusion of supplemental oxygen. The best-performing EWS systems (as evaluated on the validation dataset) include an additional score for those observation sets acquired when the patient is given supplemental oxygen support. We note, however, that the inclusion of this extra information as a marker for identifying deterioration may be problematic as different hospital units often have different protocols for using supplemental oxygen support. Secondly, the use of oxygen support is the result of a clinical intervention, which will have a direct influence on the physiological variables (certainly on SpO_2_) that are measured during that period. Although the use of oxygen support increases the ability of EWS systems to predict a major adverse event, its inclusion in a scoring system should depend on the clinical protocol employed in that hospital setting.

With the modifications described in this paper to our centile-based EWS, the performance of the proposed system is comparable to that of NEWS using all outcomes considered (Table 3, Supplemental Material – Appendix C). Our centile-based approach is based on unsupervised, statistical methodology, rather than on the supervised method used to estimate the thresholds in NEWS. Our approach can easily be extended to include new variables without having to re-train the model, and the same statistical framework can be applied to sub-populations (for example, patients with respiratory disease such as chronic obstructive pulmonary disease, COPD) or other populations (for example, paediatric patients). This unsupervised approach is a key differentiator in the methodology for constructing EWS systems, as most existing systems are optimised according to their ability to identify a compound set of patient outcomes at *T* = 24 h. Our method assumes that observations should be treated as being abnormal if they lie at the extremes of the distributions of vital signs acquired from representative sets of at-risk hospitalised patients.

There are limitations to our study. The first is that the study is based on retrospective analysis of hospitalised patients. Secondly, for the evaluation of the various EWS systems considered in this paper, we used repeated observation sets from the same patient in the analysis. This relies on the assumption that the EWS values computed from each observation set for that patient are independent (which is the usual assumption when evaluating EWS systems), and this assumption may not hold in practice. (That is, a vital-sign measurement at one point in time may be correlated with previous measurements.) However, we note that this has been the standard methodology for all the studies reported in the literature when assessing the discriminatory performance of EWS systems [1,3,4,36]. Finally, the dataset from which we derived the thresholds for our continuous CEWS score differed from the dataset with which we derived and validated the thresholds for our manual CEWS; the former was acquired from different hospitals a decade earlier. We hypothesise that differences in methods of recording respiratory rate (counting chest wall movements in manual CEWS versus automated recording of RR measurements from an electronic patient monitor in continuous CEWS) explains the differences in RR thresholds between the two scoring systems. However, we cannot exclude the possibility that other factors may provide part of the explanation.

Despite the limitations discussed above, the results of the present study are clear. Our unsupervised, data-driven approach allows for simple adjustment of the model as new representative datasets become available. Data-driven EWS systems should take into account not only the different databases used to build those systems, but also the different methods of recording the physiological variables stored in those databases.

More work is needed to establish whether systematic differences exist between vital signs recorded by patient monitors and those recorded manually. Caution needs to be exercised if early warning scores designed using databases of manually-recorded vital signs are to be used with continuously-recorded vital signs. Our statistical approach can also be used to allow the rapid development of candidate EWS systems for specific patient groups such as patients with moderate-to-severe COPD, where current early warning scores are reported to perform less well [37,38].

## Conclusion

The performance of an EWS is highly dependent on the database from which it was derived. Our unsupervised statistical approach methodology enables the rapid development of candidate EWS systems.

## Ethical approval and trial registration

Approval from the Health Research Authority was obtained for obtaining the data used in this study from the Oxford Research Ethics Committee (REC reference: 16/SC/0264).

## Funding

This publication presents independent research commissioned by the Health Innovation Challenge Fund (HICF-R9-524; WT-103703/Z/14/Z), a parallel funding partnership between the Department of Health and Wellcome Trust. The views expressed in this publication are those of the authors and not necessarily those of the Department of Health or Wellcome Trust.

## Competing interests

PJW and LT have co-developed the System for Electronic Notification and Documentation (SEND), for which Drayson Health has purchased a sole licence. The company has a research agreement with the University of Oxford and royalty agreements with Oxford University Hospitals NHS Trust and the University of Oxford. Drayson Health have paid LT consultancy fees as a member of its Strategic Advisory Board and may in the future pay PJW personal fees. DAC has been recently appointed Research Director of Drayson Health and will in future receive consultancy fees for this role.

## Appendix A Supplementary data

Checklist of STROBE/RECORD items are detailed in the attached file.
