# Supplementary material for: Manual centile-based early warning scores derived from statistical distributions of observational vital-sign data
Source: Resuscitation. 2018 Aug;129:55–60. doi: 10.1016/j.resuscitation.2018.06.003 (PMC6062656; doi:10.1016/j.resuscitation.2018.06.003)
Supplement: Supplementary file 1 [file mmc1.docx]

**Manual centile-based early warning scores derived from statistical distributions**

**of observational vital-sign data**

**Supplemental Material – Appendix**

Peter J. Watkinson, Consultant Intensive Care Physician, Nuffield Department of Clinical Neurosciences, Oxford University Hospitals NHS Trust, OX3 9DU Oxford, UK

Marco A. F. Pimentel, Postdoctoral Researcher, Institute of Biomedical Engineering, Department of Engineering Science, University of Oxford, OX3 7DQ Oxford, UK

David A. Clifton, Associate Professor of Engineering Science, Institute of Biomedical Engineering, Department of Engineering Science, University of Oxford, OX3 7DQ Oxford, UK

Lionel Tarassenko, Professor of Electrical Engineering, Institute of Biomedical Engineering, Department of Engineering Science, University of Oxford, OX3 7DQ Oxford, UK

**Correspondence to**:

Marco A. F. Pimentel, MSc., PhD.,

Institute of Biomedical Engineering,

Department of Engineering Science,

Old Road Campus Research Building,

University of Oxford,

Oxford OX3 7DQ, UK

Tel.: +44 (0) 7583 132929 | Email: [marco.pimentel@eng.ox.ac.uk](mailto:marco.pimentel@eng.ox.ac.uk)

**APPENDIX A**

In the following figures we represent the normalised histograms and cumulative distribution functions (cdf), $P(x)$, for each vital sign, computed (left column) from the vital-sign dataset collected continuously using bedside monitors (data described in Tarassenko et al.^2^); and (right column) from the training dataset which includes manual measurements of the vital sign collected electronically from patients admitted to the hospital (where *h* corresponds to the bandwidth of the Gaussian kernel). For the top plots, the central vertical line indicates the mean of the data, with the two vertical (dashed) lines either side corresponding to one standard deviation. For the lower plots, the 1^st^, 5^th^, 10^th^, 90^th^, 95^th^ and 99^th^ centiles are shown on the vertical axis and the corresponding threshold values on the horizontal axis.

**Figure A1.** Statistical distributions for HR (*h = 10*).

**Figure A2.** Statistical distributions for RR (*h = 3*).

**Figure A3.** Statistical distributions for SpO_2_ (*h = 1.5*).

**Figure A4.** Statistical distributions for Systolic BP (*h = 1*).

**Figure A5.** Statistical distributions for Temperature (manually recorded measurements, *h = 0.1*).

**APPENDIX B**

The ROC curves and precision-recall curves for all EWS systems studied (using the composite outcome of cardiac arrest, unanticipated ICU admission or death within 24 hours of an observation set) are shown in Figure B1 and Figure B2, respectively.

**Figure B1.** The receiver-operating characteristics (ROC) curve for all EWS systems studied for the combined outcome of cardiac arrest, unanticipated ICU admission or death within 24 hours of an observation set (shown in light gray). The curves for the manual CEWS (with an additional score for supplemental oxygen support) and NEWS systems are highlighted for comparison.

**Figure B2.** The precision-recall (PR) curve for all EWS systems studied for the combined outcome of cardiac arrest, unanticipated ICU admission or death within 24 hours of an observation set (shown in light gray). Precision corresponds to the Positive Predictive Value (PPV), and Recall corresponds to the True Positive Rate (or sensitivity). The curves for the manual CEWS (with an additional score for supplemental oxygen support) and NEWS systems are highlighted for comparison.

**APPENDIX C**

We conducted a sub-analysis in which we considered the performance of each EWS using each individual outcome separately. I.e., we evaluated the discriminative ability of each scoring system using cardiac arrest within 24 hours, unanticipated ICU admission within 24 hours, and in-hospital death within 24 hours.

The performance metrics for the different EWS systems on the test dataset, AUC and AUC-PR, are shown in Table C1 and Table C2, respectively. For completeness, the results obtained for the combined outcome (any of the three individual outcomes) are also shown. The AUC (95% CI) for mCEWS for in-hospital death, unanticipated ICU admission, cardiac arrest, and any of the outcomes (composite outcome, all within 24 hours, were 0.894 (0.890-0.899), 0.836 (0.830-0.842), 0.750 (0.733-0.767), and 0.868 (0.864-0.872), respectively (Table C1). For the same outcomes, the AUC (95% CI) for the other EWSs ranged from 0.772 (0.766-0.777)^39^ to 0.902 (0.898-0.906)^4^ (in-hospital death); 0.659 (0.659-0.667)^39^ to 0.824 (0.818-0.830)^3,34^ (unanticipated ICU admissions); 0.657 (0.644-0.669)^39^ to 0.764 (0.747-0.781)^4^ (cardiac arrest); 0.729 (0.725-0.734)^39^ to 0.867 (0.863-0.871)^3^ (composite outcome).

We note that for each individual outcome, the proposed approach has a similar performance to that of NEWS. Also, we observe similar results in terms of the ranking of the performance of the EWSs included in the study (considering both AUC and AUC-PR values).

In general, the performance of EWSs is lower for cardiac arrest alone, as this outcome may correspond essentially to sudden events with no preceding (noticeable) disturbance in the vital signs, which makes it less predictable (as observed previously^3^). It is also important to mention that most cardiac arrests (78.6%) are followed by in-hospital death. The finding that EWSs are poorer discriminators of unanticipated ICU admission, compared to in-hospital death, also replicates the findings of previous studies^3,36^,

Finally, we note that the comparison of the different outcomes using the AUC-PR metric is difficult due to the class imbalance problem^15,16^; i.e., the number of instances in each class (positive and negative outcome) is different when different outcomes are considered. This problem is accentuated when the outcome is the occurrence of cardiac arrest alone, in which the number of admissions with the outcome is extremely low (173). Hence, this metric can be of limited use when evaluating the performance of this systems when the events are very rare.

**Table C1.** Area under the receiver-operating characteristics curve (AUC) and corresponding 95% confidence interval (CI) for the Manual centile-based EWS (CEWS) and 22 other early warning score (EWS) systems, using cardiac arrest within 24 hours, unanticipated admission to ICU within 24 hours, in-hospital death within 24 hours, or any of the three (composite outcome) within 24 hours of an observation set. The EWS number (EWS no.) refers to those used in other figures and sections of the manuscript. Results are presented in descending order of AUC for the composite outcome, with the results relating to the scores that use the methodology discussed in the manuscript highlighted. [*] indicates systems that have an additional score for supplemental oxygen support.

| EWS no. | EWS | Composite outcome | In-hospital death | Unanticipated ICU admission |  | Cardiac arrest |
| --- | --- | --- | --- | --- | --- | --- |
| - | **Manual CEWS [*]** | **0.868**  **(0.864 - 0.872)** | **0.894**  **(0.890 - 0.899)** | **0.836**  **(0.830 - 0.842)** |  | **0.750**  **(0.733 - 0.767)** |
| 21 | NEWS^3^ [*] | 0.867  (0.863 - 0.871) | 0.901  (0.897 - 0.905) | 0.824  (0.818 - 0.830) |  | 0.760  (0.744 - 0.777) |
| 22 | Badriyah et al. (2014)^4^ [*] | 0.865  (0.861 - 0.869) | 0.902  (0.898 - 0.906) | 0.817  (0.810 - 0.824) |  | 0.764  (0.747 - 0.781) |
| 19 | Lilienfeld-Toal et al. (2007)^34^ [*] | 0.860  (0.857 - 0.864) | 0.886  (0.881 - 0.890) | 0.824  (0.818 - 0.830) |  | 0.740  (0.722 - 0.757) |
| 18 | Lilienfeld-Toal et al. (2007)^34^ | 0.846  (0.842 - 0.850) | 0.875  (0.870 - 0.879) | 0.807  (0.800 - 0.813) |  | 0.729  (0.712 - 0.746) |
| 8 | Goldhill et al. (2005)^25^ | 0.846  (0.842 - 0.850) | 0.875  (0.870 - 0.879) | 0.806  (0.800 - 0.813) |  | 0.728  (0.711 - 0.745) |
| 11 | Paterson et al. (2006)^28^ | 0.843  (0.839 - 0.847) | 0.871  (0.866 - 0.876) | 0.804  (0.798 - 0.811) |  | 0.726  (0.709 - 0.743) |
| - | **Manual CEWS** | **0.836**  **(0.832 - 0.840)** | **0.860**  **(0.855 - 0.865)** | **0.805**  **(0.799 - 0.811)** |  | **0.720**  **(0.702 - 0.737)** |
| 7 | Allen (2004)^24^ | 0.827  (0.823 - 0.831) | 0.845  (0.840 - 0.850) | 0.801  (0.795 - 0.807) |  | 0.697  (0.679 - 0.714) |
| 9 | Chatterjee et al. (2005)^26^ | 0.826  (0.822 - 0.830) | 0.841  (0.836 - 0.846) | 0.806  (0.800 - 0.813) |  | 0.702  (0.684 - 0.720) |
| 1 | Wright et al. (2000)^18^ | 0.823  (0.818 - 0.827) | 0.844  (0.839 - 0.849) | 0.794  (0.787 - 0.800) |  | 0.703  (0.685 - 0.720) |
| 4 | Cooper et al. (2001)^21^ | 0.823  (0.818 - 0.827) | 0.845  (0.840 - 0.850) | 0.793  (0.786 - 0.799) |  | 0.705  (0.687 - 0.722) |
| 5 | Subbe et al. (2003)^22^ | 0.822  (0.818 - 0.826) | 0.844  (0.838 - 0.849) | 0.794  (0.787 - 0.800) |  | 0.703  (0.685 - 0.720) |
| 3 | Riley et al. (2001)^20^ | 0.821  (0.817 - 0.825) | 0.842  (0.837 - 0.847) | 0.793  (0.787 - 0.800) |  | 0.702  (0.685 - 0.720) |
| 2 | Subbe et al. (2001)^19^ | 0.821  (0.817 - 0.825) | 0.842  (0.837 - 0.847) | 0.793  (0.786 - 0.799) |  | 0.703  (0.685 - 0.720) |
| 12 | Smith et al. (2006)^35^ | 0.821  (0.817 - 0.825) | 0.843  (0.838 - 0.848) | 0.792  (0.785 - 0.798) |  | 0.701  (0.684 - 0.719) |
| 10 | Andrews et al. (2005)^27^ | 0.818  (0.813 - 0.822) | 0.846  (0.841 - 0.851) | 0.780  (0.773 - 0.786) |  | 0.715  (0.697 - 0.732) |
| 14 | Gardner-Thorpe et al. (2006)^30^ | 0.817  (0.813 - 0.822) | 0.846  (0.841 - 0.851) | 0.779  (0.773 - 0.786) |  | 0.715  (0.698 - 0.732) |
| 13 | Lam et al. (2006)^29^ | 0.817  (0.813 - 0.822) | 0.838  (0.833 - 0.843) | 0.790  (0.783 - 0.796) |  | 0.701  (0.683 - 0.718) |
| 16 | Odell (2007)^32^ | 0.817  (0.813 - 0.822) | 0.846  (0.841 - 0.851) | 0.779  (0.773 - 0.786) |  | 0.715  (0.698 - 0.733) |
| 17 | Hancock et al. (2007)^33^ | 0.817  (0.813 - 0.821) | 0.837  (0.832 - 0.842) | 0.789  (0.783 - 0.796) |  | 0.700  (0.683 - 0.718) |
| 6 | Rees et al. (2004)^23^ | 0.815  (0.811 - 0.820) | 0.839  (0.834 - 0.844) | 0.785  (0.778 - 0.791) |  | 0.707  (0.690 - 0.724) |
| **20** | **Continuous CEWS^2^** | **0.808**  **(0.804 - 0.812)** | **0.833**  **(0.827 - 0.838)** | **0.777**  **(0.770 - 0.783)** |  | **0.689**  **(0.671 - 0.707)** |
| 15 | Subbe et al. (2007)^31^ | 0.768  (0.764 - 0.773) | 0.824  (0.818 - 0.829) | 0.697  (0.690 - 0.704) |  | 0.681  (0.664 - 0.699) |
| - | CART, Churpek et al. (2012)^39^ | 0.729  (0.725 – 0.734) | 0.772  (0.766 – 0.777) | 0.659  (0.651 – 0.667) |  | 0.657  (0.644 – 0.669) |

**Table B2.** Area under the precision-recall curve (AUC-PR) and corresponding 95% confidence interval (CI) for the Manual centile-based EWS (CEWS) and 22 other early warning score (EWS) systems, using cardiac arrest within 24 hours, unanticipated admission to ICU within 24 hours, in-hospital death within 24 hours, or any of the three (composite outcome) within 24 hours of an observation set. The EWS number (EWS no.) refers to those used in other figures and sections of the manuscript. Results are presented in descending order of AUC for the composite outcome, with the results relating to the scores that use the methodology discussed in the manuscript highlighted. [*] indicates systems that have an additional score for supplemental oxygen support.

| EWS no. | EWS | Composite outcome | In-hospital death | Unanticipated ICU admission | Cardiac arrest |
| --- | --- | --- | --- | --- | --- |
| - | **Manual CEWS [*]** | **0.161**  **(0.155 - 0.167)** | **0.142**  **(0.135 - 0.149)** | **0.043**  **(0.038 - 0.048)** | **0.003**  **(0.000 - 0.007)** |
| 21 | NEWS^3^ [*] | 0.163  (0.157 - 0.169) | 0.154  (0.147 - 0.162) | 0.035  (0.030 - 0.039) | 0.003  (0.000 - 0.006) |
| 22 | Badriyah et al. (2014)^4^ [*] | 0.157  (0.151 - 0.162) | 0.153  (0.146 - 0.161) | 0.031  (0.027 - 0.035) | 0.003  (0.000 - 0.006) |
| 19 | Lilienfeld-Toal et al. (2007)^34^ [*] | 0.151  (0.146 - 0.157) | 0.135  (0.128 - 0.142) | 0.036  (0.032 - 0.041) | 0.003  (0.000 - 0.006) |
| 18 | Lilienfeld-Toal et al. (2007)^34^ | 0.148  (0.142 - 0.153) | 0.131  (0.124 - 0.138) | 0.036  (0.031 - 0.040) | 0.003  (0.000 - 0.006) |
| 8 | Goldhill et al. (2005)^25^ | 0.143  (0.137 - 0.148) | 0.128  (0.121 - 0.135) | 0.033  (0.029 - 0.038) | 0.003  (0.000 - 0.006) |
| 11 | Paterson et al. (2006)^28^ | 0.159  (0.153 - 0.164) | 0.139  (0.132 - 0.146) | 0.039  (0.034 - 0.044) | 0.003  (0.000 - 0.007) |
| - | **Manual CEWS** | **0.140**  **(0.135 - 0.146)** | **0.117**  **(0.111 - 0.124)** | **0.037**  **(0.033 - 0.042)** | **0.003**  **(0.000 - 0.006)** |
| 7 | Allen (2004)^24^ | 0.124  (0.118 - 0.129) | 0.100  (0.094 - 0.107) | 0.035  (0.031 - 0.040) | 0.003  (0.000 - 0.006) |
| 9 | Chatterjee et al. (2005)^26^ | 0.130  (0.125 - 0.135) | 0.094  (0.088 - 0.100) | 0.044  (0.039 - 0.049) | 0.003  (0.000 - 0.006) |
| 1 | Wright et al. (2000)^18^ | 0.129  (0.124 - 0.135) | 0.094  (0.088 - 0.100) | 0.043  (0.038 - 0.048) | 0.003  (0.000 - 0.006) |
| 4 | Cooper et al. (2001)^21^ | 0.129  (0.124 - 0.134) | 0.094  (0.088 - 0.100) | 0.043  (0.038 - 0.048) | 0.003  (0.000 - 0.006) |
| 5 | Subbe et al. (2003)^22^ | 0.130  (0.125 - 0.136) | 0.095  (0.089 - 0.101) | 0.043  (0.038 - 0.048) | 0.003  (0.000 - 0.006) |
| 3 | Riley et al. (2001)^20^ | 0.126  (0.120 - 0.131) | 0.090  (0.085 - 0.096) | 0.043  (0.038 - 0.047) | 0.003  (0.000 - 0.006) |
| 2 | Subbe et al. (2001)^19^ | 0.123  (0.118 - 0.128) | 0.091  (0.085 - 0.097) | 0.040  (0.035 - 0.045) | 0.003  (0.000 - 0.006) |
| 12 | Smith et al. (2006)^35^ | 0.130  (0.124 - 0.135) | 0.094  (0.088 - 0.100) | 0.043  (0.038 - 0.048) | 0.003  (0.000 - 0.006) |
| 10 | Andrews et al. (2005)^27^ | 0.131  (0.126 - 0.137) | 0.101  (0.094 - 0.107) | 0.041  (0.036 - 0.046) | 0.003  (0.000 - 0.006) |
| 14 | Gardner-Thorpe et al. (2006)^30^ | 0.130  (0.125 - 0.136) | 0.099  (0.092 - 0.105) | 0.041  (0.036 - 0.046) | 0.003  (0.000 - 0.006) |
| 13 | Lam et al. (2006)^29^ | 0.127  (0.122 - 0.133) | 0.096  (0.090 - 0.102) | 0.042  (0.037 - 0.046) | 0.003  (0.000 - 0.006) |
| 16 | Odell (2007)^32^ | 0.130  (0.125 - 0.136) | 0.099  (0.093 - 0.105) | 0.041  (0.036 - 0.046) | 0.003  (0.000 - 0.006) |
| 17 | Hancock et al. (2007)^33^ | 0.128  (0.122 - 0.133) | 0.096  (0.090 - 0.103) | 0.041  (0.037 - 0.046) | 0.003  (0.000 - 0.006) |
| 6 | Rees et al. (2004)^23^ | 0.128  (0.123 - 0.133) | 0.097  (0.091 - 0.103) | 0.041  (0.036 - 0.046) | 0.003  (0.000 - 0.006) |
| **20** | **Continuous CEWS^2^** | 0.128  (0.123 - 0.133) | 0.109  (0.103 - 0.116) | 0.033  (0.029 - 0.037) | 0.003  (0.000 - 0.006) |
| 15 | Subbe et al. (2007)^31^ | 0.101  (0.096 - 0.106) | 0.094  (0.088 - 0.100) | 0.023  (0.019 - 0.026) | 0.003  (0.000 - 0.006) |
| - | CART, Churpek et al. (2012)^39^ | 0.098  (0.092 – 0.104) | 0.091  (0.085 – 0.096) | 0.021  (0.016 – 0.025) | 0.003  (0.000 - 0.006) |
