# Supplementary material for: Manual centile-based early warning scores derived from statistical distributions of observational vital-sign data
Source: Resuscitation. 2018 Aug;129:55–60. doi: 10.1016/j.resuscitation.2018.06.003 (PMC6062656; doi:10.1016/j.resuscitation.2018.06.003)

Figure A1. Statistical distributions for heart rate, HR.

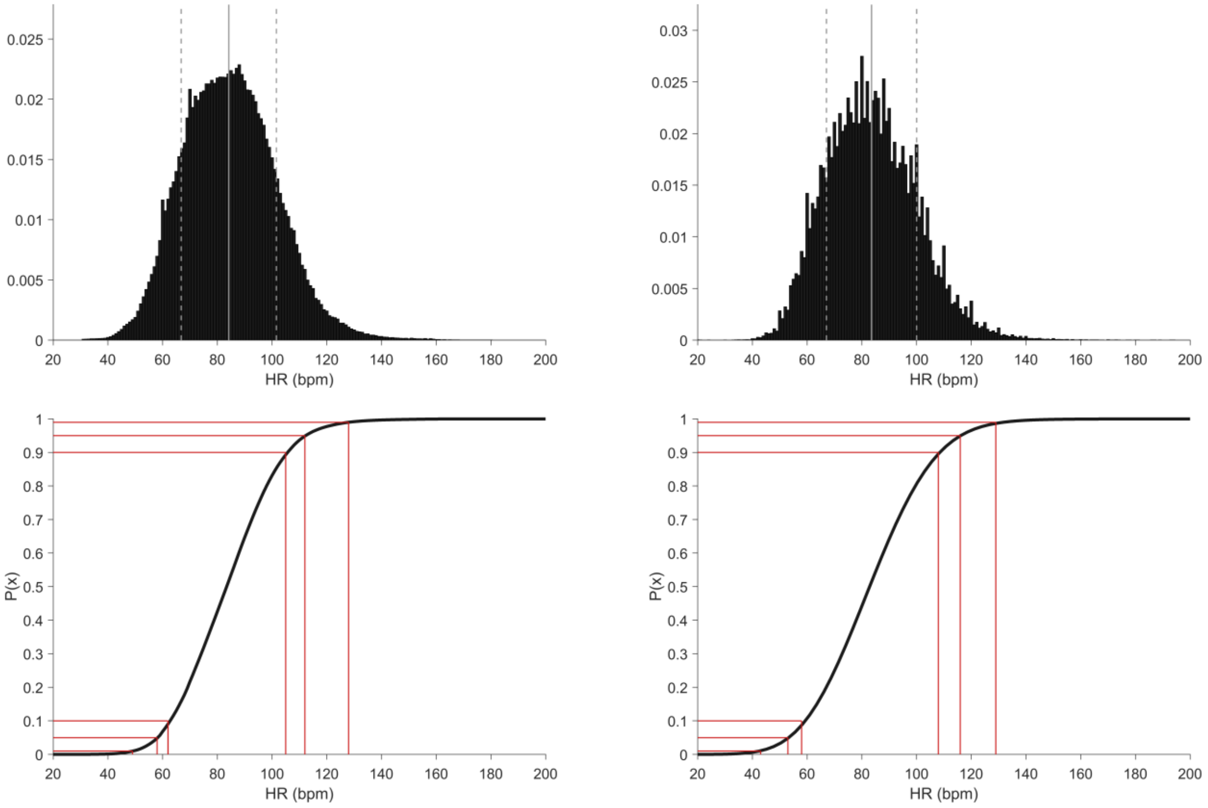

Figure A2. Statistical distributions for RR.

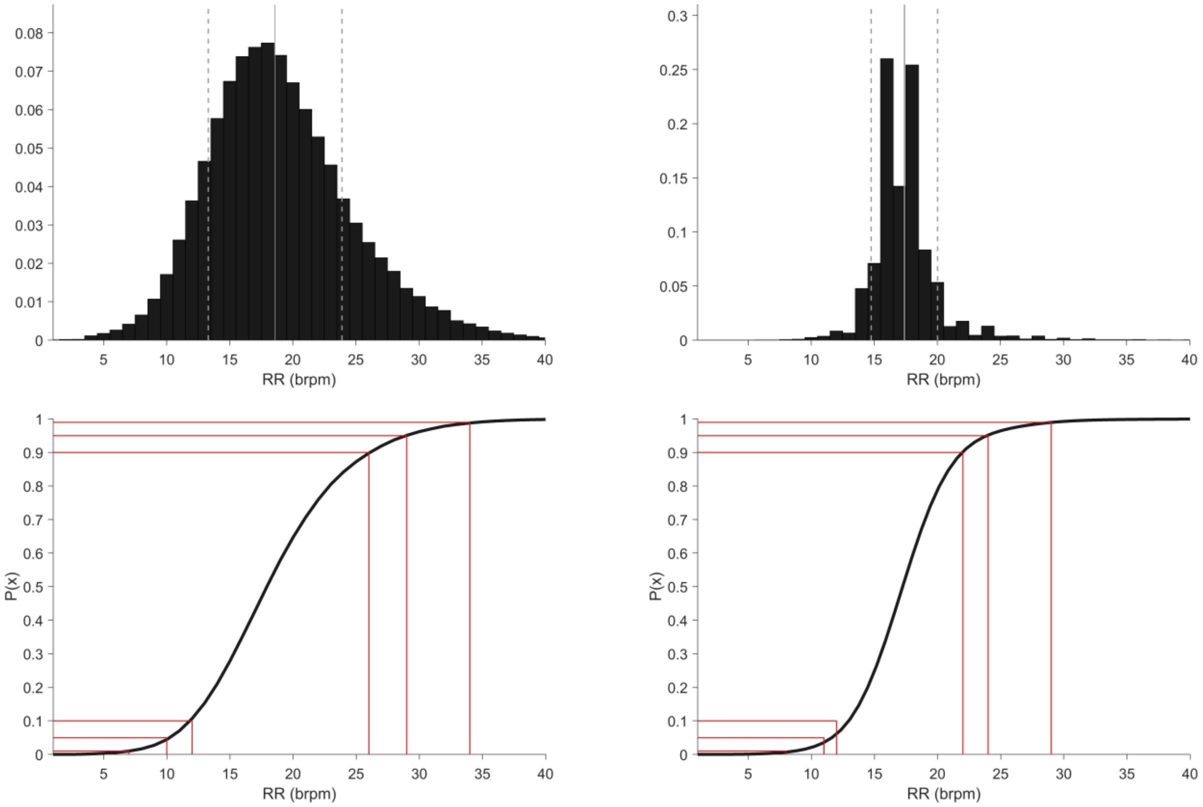

Figure A3. Statistical distributions for SpO_2.

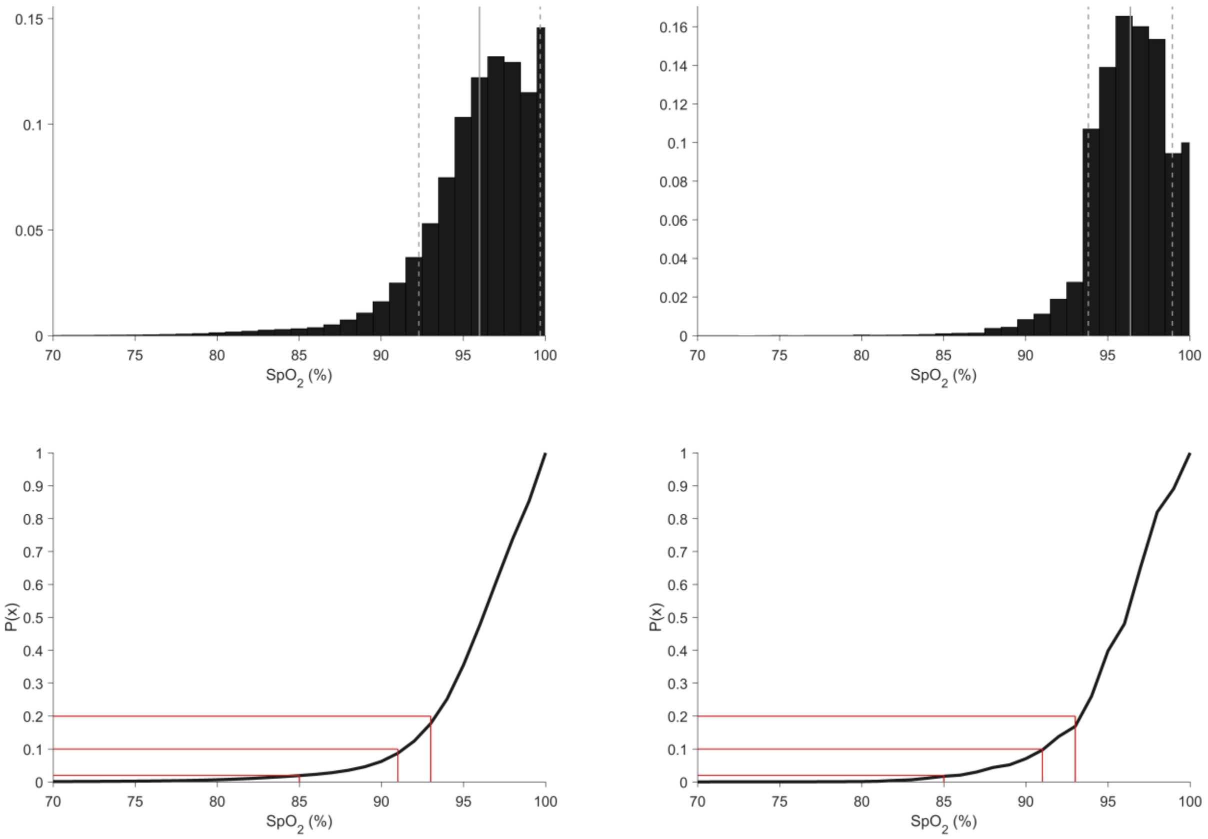

Figure A4. Statistical distributions for Systolic BP.

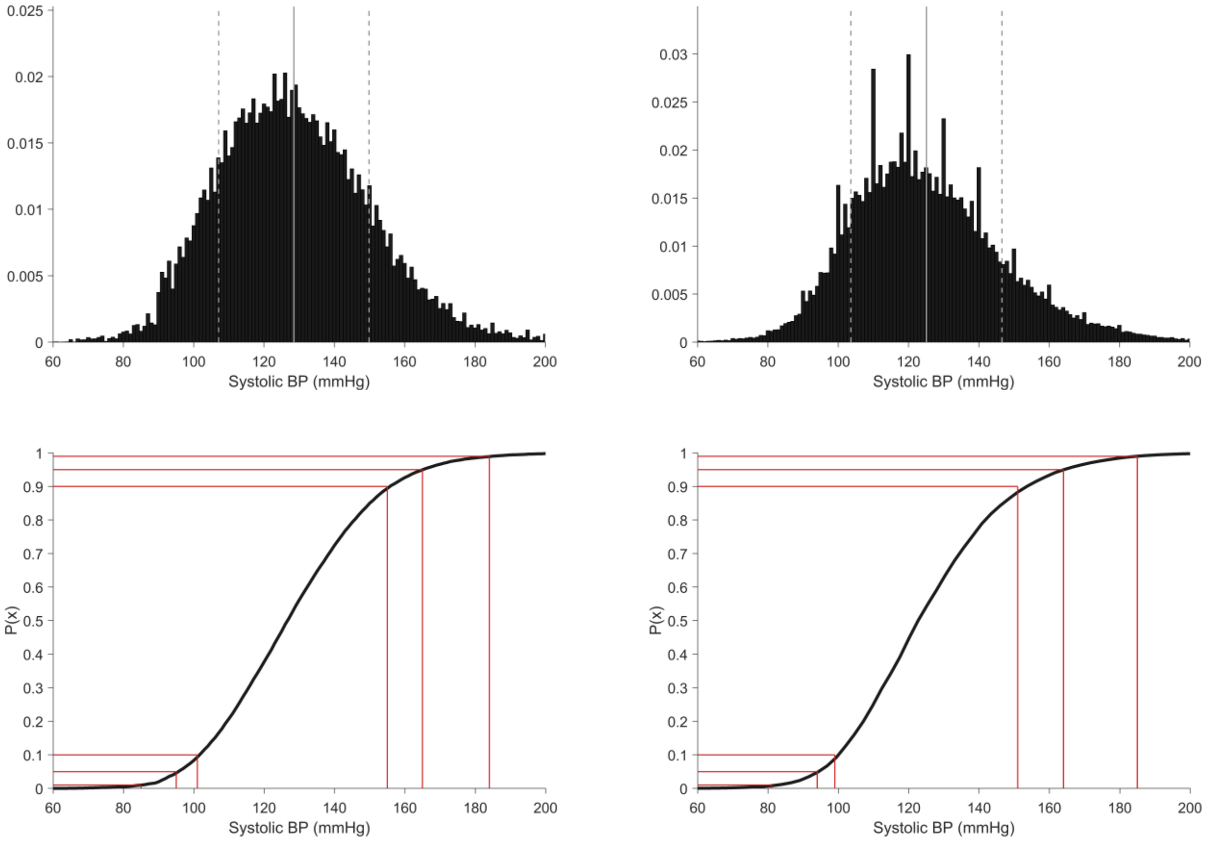

Figure A5. Statistical distributions for Temperature (based on manually-recorded measurements).

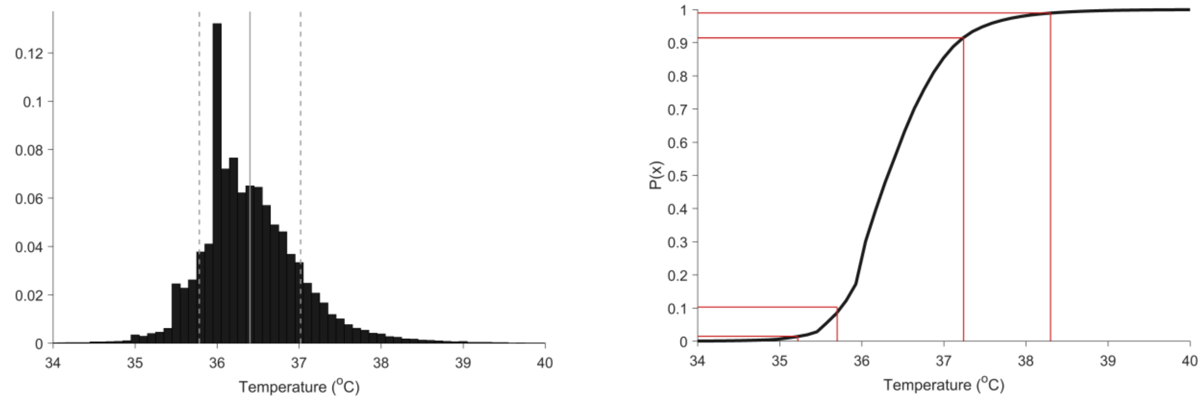

Figure B1. The receiver-operating characteristics (ROC) curve for all EWS systems studied for the combined outcome of cardiac arrest, unanticipated ICU admission or death within 24 hours of an observation set (shown in light gray). The curves for the manual CEWS (with an additional score for supplemental oxygen support) and NEWS systems are highlighted for comparison.

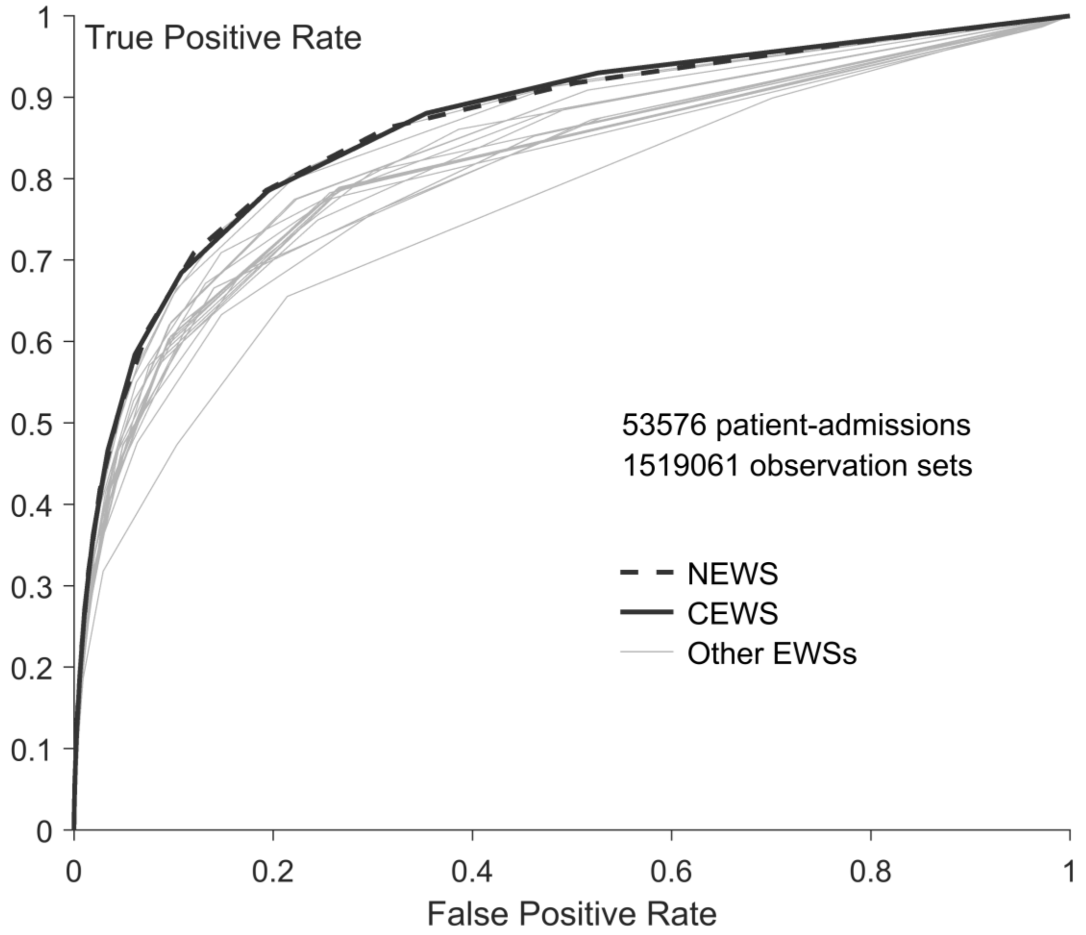

Figure B2. The precision-recall (PR) curve for all EWS systems studied for the combined outcome of cardiac arrest, unanticipated ICU admission or death within 24 hours of an observation set (shown in light gray). Precision corresponds to the Positive Predictive Value (PPV), and Recall corresponds to the True Positive Rate (or sensitivity). The curves for the manual CEWS (with an additional score for supplemental oxygen support) and NEWS systems are highlighted for comparison.

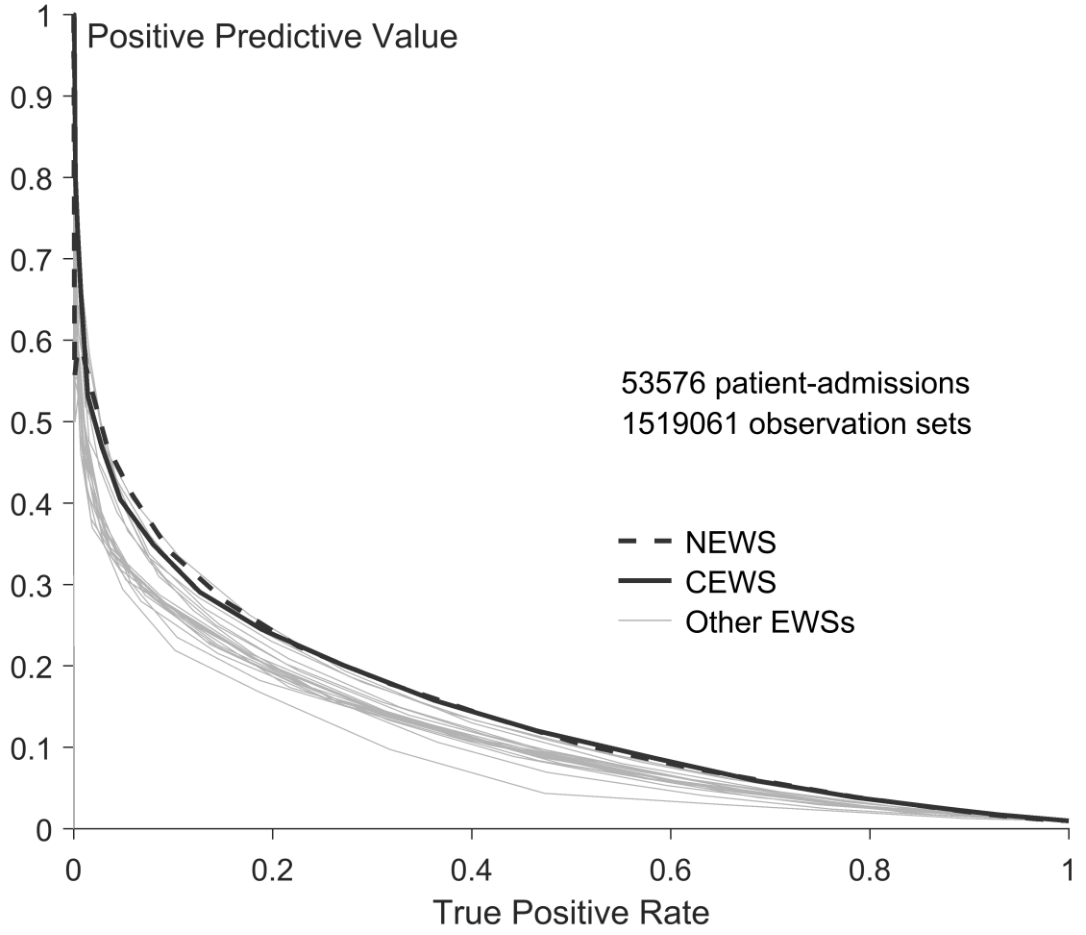

Supplement: Supplementary file 2 [file mmc2.pdf]
